# Case of Perforation of the Stomach

**Published:** 1825-04

**Authors:** J. R. Griffiths


### Art. III.—*Case of Perforation of the Stomach.*
By J. R. Griffiths, Esq.

Though similar cases to the following have been often described and reasoned upon, yet I think the relation of it may serve to put medical men in mind that such do occur occasionally, and lead them to make a cautious prognosis in those resembling it.

It does not appear that there is any real diagnostic symptom of the disease; but possibly, in another case, the previous history might lead one to suspect it, and then, perhaps, it would be more proper to administer medicines by the rectum instead of the mouth: not that there is much chance of being of any service; but we should avoid increasing the inflammation of the peritoneum, by giving stimuli which might escape into the abdomen.

On Sunday, the 6th instant, Mary Ann Underhill, a very fine young woman, aged twenty-two, after making an unusually hearty breakfast of fat bacon, was attacked with pain in the stomach, and vomited some of the food she had taken. She was retiring to her own room, when the pain became so acute that it deprived her of power to call for assistance, but the family were soon alarmed by her screams. She was found upon her hands and knees on the floor, suffering great agony: she complained of intense pain in the lower part of the belly, which she said appeared to draw her together. She was assisted into bed; and the pain continued to increase to such a degree, that she tore her hair, beat every thing about her, and seemed insensible to all but the intensity of her sufferings.

About an hour after she was attacked, she was seen by my friend, Dr. Ferguson, and myself. We found her in a state of extreme suffering; she was only just able to say she was in horrible pain, and to pray earnestly for something to relieve her. The muscles of the abdomen were contracted in knots; pulse not the least affected.

We imagined it to be a violent spasmodic attack, most probably hysteric colic. By way of assisting the action of the medicines employed, we took a few ounces of blood from the arm, gave her large doses of æther and laudanum, and fomented the abdomen with hot water.

This plan was continued for some time, without producing any effect, but towards evening she gradually became better; but still complained of great soreness of the abdomen, and pain in her sides and shoulders, accompanied with much thirst: pulse still natural.

We saw her at eleven P.M. She still continued to get better; had been well enough to enter into conversation with those about her, and expressed her conviction that she should die suddenly. She was then left with a person to sit up by her during the night; and we expected that she would feel nothing in the morning but some degree of soreness.

She remained much in the same state, occasionally sitting up to take some tea, and conversing with her nurse; but she was unable to procure any sleep. About four o'clock she complained of an increase of pain: more laudanum was given, which afforded some relief. As she continued free from much suffering, her attendant ventured to leave her at seven; but returned in about half an hour, when she found a very great change had taken place, and sent for me. On my arrival, I found her in *articulo mortis*, and by nine she was dead.

It since appears that she had not been well for the last two months: there was a most unaccountable indolence about her, and she would sit for hours by the fire-side, and say she felt certain she had some disease which nobody understood, and that she should die suddenly; and expressed a wish that she might be opened, for the benefit of others. Her fellow-servant states that she was frequently sick after eating; and, when making the beds, would sometimes scream out, and throw herself forward, saying she had violent pain in the stomach. It is worthy of remark, that she had gained flesh during these two months.

On the day of her death, I saw my friend, Mr. Shaw, and described the case to him, expressing a wish that he would examine the body with me. As soon as he had heard my account, he at once said he thought it rupture of the stomach.

Upon opening the abdomen, the stomach was found to be perforated in two places: one hole was on the anterior surface, nearly in the centre, but rather towards the pylorus; the other on the posterior, a little nearer the cardiac orifice; both were circular. The posterior one, about the size of a shilling, looked much as if it had been made with a punch, all the coats being destroyed to the same extent. The anterior had an opening in the peritoneum, about the size of the top of the little finger; but the other coats were destroyed to the same extent as the posterior orifice. The rest of the stomach was free from inflammation, or disease of any kind. The peritoneum was inflamed, from the contents of the stomach having escaped into the abdomen.

***Bentinck-street; February 19th, 1825.***

